# Brain functional changes following spinal manipulation therapy in patients with lumbar disc herniation and chronic low back pain: a scoping review

**DOI:** 10.3389/fneur.2025.1712320

**Published:** 2026-01-06

**Authors:** Lizhen Cao, Jing Shu, Bibao Li

**Affiliations:** 1 Department of Nursing, Hubei University of Chinese Medicine, Wuhan, China; 2 Hubei Provincial Hospital of Traditional Chinese Medicine, Wuhan, China

**Keywords:** brain mechanisms, fMRI, lumbar disc herniation, pain, spinal manipulation therapy

## Abstract

**Purpose:**

Chronic low back pain (LBP) is a common symptom in patients with lumbar disc herniation (LDH), and spinal manipulation-related therapies can alleviate pain. This study aimed to review the clinical research evidence regarding the central nervous mechanisms of spinal manipulation in treating chronic lower back pain related to lumbar disc herniation, summarize the current state of research, identify existing gaps, and lay the foundation for future studies.

**Methods:**

This scoping review was conducted according to established methodological frameworks. Original studies were retrieved from eight databases from inception to August 1, 2025. Of the 169 articles, 10 were ultimately selected as meeting the inclusion criteria. The data were systematically organized and categorized according to the research objectives.

**Results:**

The ten included studies demonstrated that spinal manipulation therapies induce significant changes in brain activity and connectivity. Key findings include modulation of the prefrontal cortex, visual network, and default mode network. These neuroplastic changes were correlated with improvements in clinical pain intensity (VAS), functional disability (ODI, JOA), and emotional states (SAS, SDS). Acupoint-specific manipulation was found to elicit distinct brain activation patterns compared to non-acupoint stimulation.

**Conclusion:**

Preliminary findings indicate that spinal manipulation-related therapies may alleviate LDH-associated chronic low back pain and mood disorders by modulating regions such as the prefrontal cortex, the visual network, and the default mode network. The development of standardized clinical practices and clarification of central nervous mechanisms are vital for improving pain management in this population group.

**Systematic review registration:**

https://www.crd.york.ac.uk/PROSPERO/view/CRD420251109911, CRD420251109911.

## 1 Introduction

Lumbar disc herniation (LDH) is a common cause of chronic low back pain (CLBP) (1, 2). Clinically, it is characterized by the localized displacement of the nucleus pulposus within the intervertebral disc, resulting in nerve root compression and causing somatic pain and radicular symptoms. In the United States, the prevalence of LDH is 1–3% of the general population (3), with a lifetime prevalence as high as 70% (4, 5), making it a leading cause of disability (6). The risk of developing LDH increases with age (7), and recent epidemiological trends have shown that its incidence is also rising among younger populations (8, 9). Owing to its often protracted course of treatment, LDH is a major source of CLBP (10). Chronic low back pain associated with LDH is also closely related to functional disability, work absenteeism, and reduced quality of life (11). Patients with LDH-related CLBP experience persistent pain and commonly suffer from associated negative emotional states, including anxiety, depression, pain catastrophizing, and fear-avoidance behaviors. One study found that among patients with LDH and CLBP, 33.3% had comorbid depression and 37.6% had comorbid anxiety (12). Typically, only 10–20% of patients who do not respond to conservative treatment eventually require surgical intervention (13, 14).

Spinal manipulation therapy (SMT) is a non-surgical physical intervention in which specific manipulative techniques are performed on the musculoskeletal structure by trained professionals. In China, SMT is often practiced in the form of Tuina, a massage therapy rooted in traditional Chinese medicine theory, and is therefore widely used throughout the country. Multiple randomized controlled trials (RCTs) have confirmed the efficacy of SMT in the treatment of LDH (15, 16). A meta-analysis involving 13,075 patients further indicated that SMT is more effective than traction and traditional Chinese medicine for treating LDH (17). Traditionally, it was believed that CLBP caused by LDH stems entirely from the mechanical compression of nerve roots by herniated disc material. Therefore, SMT primarily achieves pain relief by restoring the paraspinal muscle function and promoting blood circulation (18). However, the pain-relieving theory of SMT alleviating the local compression of LDH is controversial. A considerable number of patients continue to experience chronic pain and negative emotions even after the compressive pathology has been resolved (19), indicating that, beyond peripheral nerve injury, central nervous system (CNS) mechanisms also play a role in maintaining chronic pain and related emotional disorders (20). Pickar was the first to propose a theoretical framework in which SMT can affect the central nervous system (21). Previous research suggests that the perception of chronic pain is intricately linked to brain function activation, with the brain’s high level of signal integration ultimately responsible for processing the pain signals (22, 23). Extensive research has demonstrated that SMT induces a series of neurochemical responses in the central and peripheral nervous systems (24–27). Research indicates that mechanical stimulation of the lower back leads to a notable increase in activity in various brain regions (28). One study observed emotional changes in patients with lower back pain and found that after just one session of SMT, patients experienced a decrease in their fear of movement (29). Within the CNS, central sensitization, characterized by heightened responsiveness to persistent noxious input, plays a key role. Moreover, structural and functional reorganization of the brain is involved in the pathophysiology of chronic pain (30).

Functional magnetic resonance imaging (fMRI) is a technique that obtains images by measuring blood oxygen levels and changes in blood flow in various regions of the brain. In recent years, blood oxygen level-dependent (BOLD) fMRI technology has made breakthrough advances, making it possible to non-invasively identify changes in brain activity, including alterations in spontaneous neural activity in specific brain regions and their associations with physiological states. fMRI mainly includes two types of assessments: task-state functional magnetic resonance imaging (T-fMRI) and resting-state functional magnetic resonance imaging (rs-fMRI). rs-fMRI provides critical insights into the biological characteristics of the central nervous system in patients with LDH. Emerging evidence shows that (31), compared to healthy controls, patients with LDH exhibit changes in the functional activity of specific brain regions, which may mediate the association between chronic pain and depression. Based on these findings, modern studies have proposed the theory of “brain-bone crosstalk” (32, 33). This theory posits that LDH-related CLBP and negative emotions involve a complex bidirectional dynamic process that includes neuropathological changes and CNS neuroplasticity, rather than a simple linear causal relationship. Rupture of the annulus fibrosus and herniation of the nucleus pulposus can compress nearby nerve roots, leading to lower back pain and functional impairment (34). Simultaneously, the immune system detects the exposed nucleus pulposus and releases various proinflammatory factors, which heighten both the sensitivity and intensity of pain (35). Negative emotions, such as anxiety and depression, can intensify pain, potentially worsening the severity and duration of chronic pain (36, 37). LDH is often accompanied by depression, particularly in women experiencing severe pain and a prolonged illness (38). The limited understanding of how SMT modulates brain function in patients with LDH has hindered the development of the most effective treatment methods for LDH-related CLBP. However, few studies have explored changes in brain structure and function after spinal manipulation in patients with LDH using fMRI. This study primarily reviews existing research on resting-state fMRI in patients with LDH following spinal manipulation therapy, analyzing the changes in brain connectivity and function before and after the intervention.

This review summarizes the regulatory effects of SMT on brain functional connectivity and activity in patients with LDH and examines the CNS mechanisms underlying the efficacy of SMT. This not only offers theoretical support for clinical practice but also establishes a foundation for future research on the neural mechanisms underlying LDH. Currently, most research on manual therapy for LDH emphasizes peripheral mechanisms, such as paraspinal muscle function recovery and local blood supply improvement, while the exploration of central nervous system mechanisms remains limited. However, chronic low back pain in patients with LDH is closely linked to CNS and brain functional network reconstruction, making it clinically and scientifically valuable to explore the central nervous effects of SMT. In the future, LDH treatment and research should focus on the CNS changes induced by SMT, using these functional changes as a core evaluation indicator, promoting the standardization of SMT procedures, and ultimately providing evidence-based support for precise pain management in LDH.

## 2 Methods

This scoping review adheres to The Preferred Reporting Items for Systematic reviews and Meta-Analyses extension for Scoping Reviews (PRISMA-ScR) Checklist, and the Arksey and O’Malley framework were used to conduct this review (39, 40).

### 2.1 Search strategy

We systematically searched eight databases: China National Knowledge Infrastructure (CNKI), Wanfang Data, Chinese Biomedical Literature Database (CBM), Chongqing VIP Information (VIP), PubMed, Web of Science (WOS), EMBASE, and the Cochrane Library. Our search original research in both Chinese and English published from the inception of each database until August 1, 2025. Focusing on the subjects of “lumbar disc herniation,” “pain,” “Functional magnetic resonance imaging,” and “musculoskeletal manipulation,” we conducted keyword searches in each database, combining Medical Subject Headings and free text terms. Supplementary material provides a detailed description of the OSF pre-registered ID and the complete retrieval strategy used in all databases.

### 2.2 Inclusion criteria

#### 2.2.1 Study type

This review included all original studies on spinal manipulation therapy for chronic lower back pain related to lumbar disc herniation, including randomized controlled trials, non-randomized controlled trials, case–control studies, cohort studies, and case reports. Animal studies, unpublished abstracts, review articles, conference proceedings, guidelines, and other review literature were excluded.

#### 2.2.2 Study participants

Eligible participants were patients diagnosed with lumbar disc herniation based on imaging or established guidelines. All participants were required to have chronic lower back pain, with no restrictions on pain type. Patients with other comorbidities or symptoms were excluded from the study.

#### 2.2.3 Interventions and control

The intervention group received spinal manipulation therapy, including specific manipulation techniques. These therapies can be used alone or in combination with other manual or non-manual therapies. The control group could involve a variety of treatments, such as no treatment, sham therapy, different types of manual therapies, medication, or rehabilitation. Studies were excluded if spinal manipulation was not the sole intervention variable.

#### 2.2.4 Outcome indicators

Studies were required to report the participants’ functional magnetic resonance imaging (fMRI) outcomes.

### 2.3 Literature screening and data extraction

All records were imported into the Covidence electronic platform, and duplicates were eliminated. Two reviewers (LC and BL) independently screened the titles and abstracts, followed by a full-text screening. Any discrepancies during the screening process were discussed and resolved by a third reviewer (JS). Studies meeting the inclusion criteria were subjected to data extraction.

Data extraction was independently performed by one researcher (LC) and checked for accuracy by two others (BL and JS). We developed a data extraction template to collect the following information: study characteristics (author, year of publication, country, and study design), participant characteristics (including group assignment, pain duration, intervention method, and duration), fMRI assessment, study conclusions.

## 3 Results

### 3.1 Literature search

**Figure 1:**
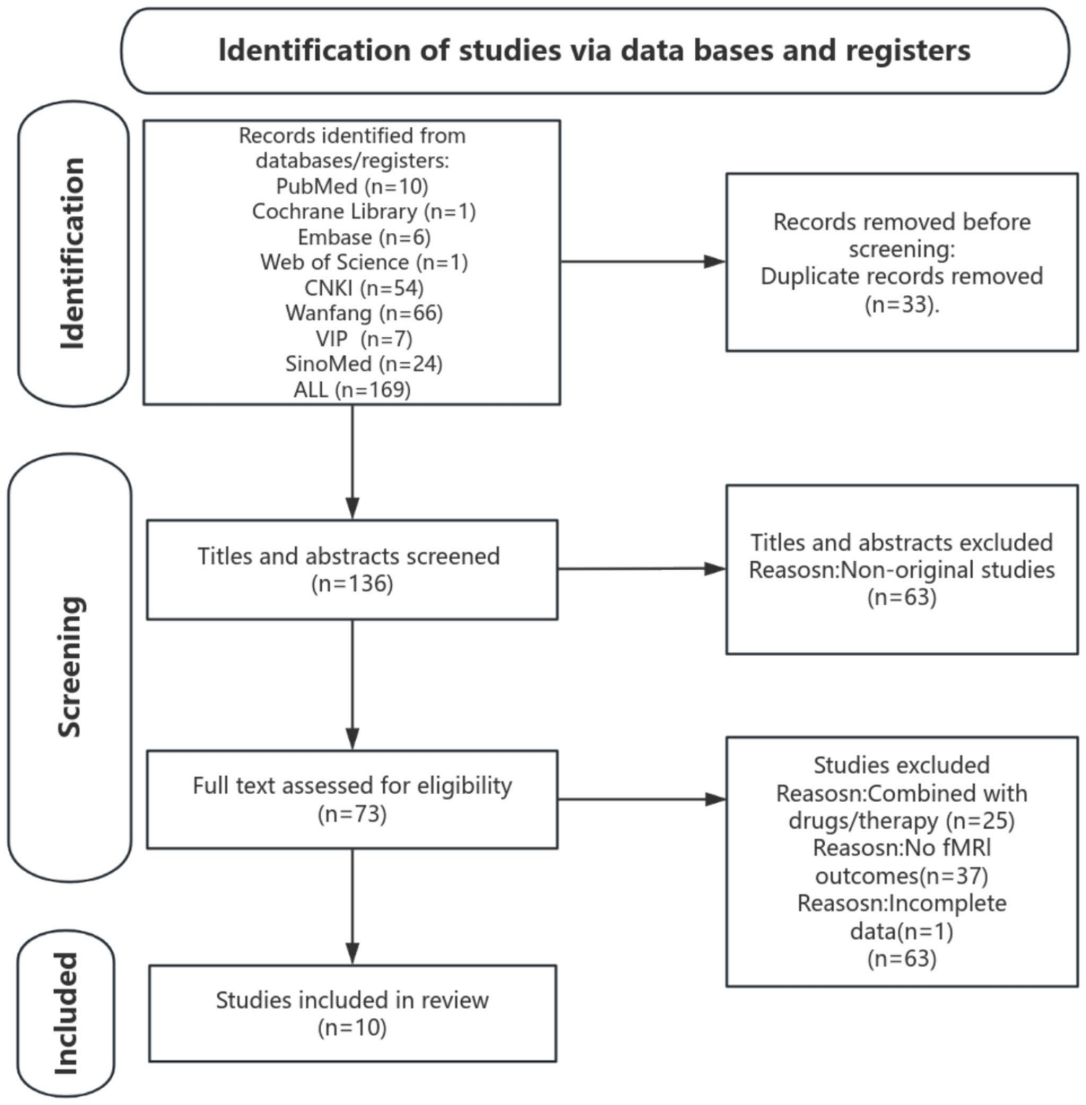
PRISMA flow diagram.

A total of 169 articles were identified by searching eight Chinese and English databases, of which 10 articles met the inclusion criteria. The study screening process followed the Preferred Reporting Items for Systematic Reviews and Meta-Analyses (PRISMA) guidelines, as illustrated in Figure 1. Table 1 provides a detailed overview of the characteristics of the included study.

**Table 1:** A comprehensive overview of the study characteristics and key findings of the fully included studies.

Authors and year	Participants characteristics	Sample size	Intervention method	fMRI parameters	Results
Yuan et al. (2015) (41)	Lumbar disc herniation, disease duration >6 months Inclusion criteria: 25–55 years old; right-handed; lumbar disc herniation confirmed by MRI; average low back pain VAS ≥ 30/100; ODI functional disability index ≥20%; has not recently taken painkillers, neurotrophic drugs, or sedatives, and has not undergone systematic treatment Average age: 40.5 ± 10.3 years Average disease duration: 19.0 ± 7.7 months	8 cases: 3 males and 5 females	Spinal manipulation therapy, once every 3 days, for a total of 6 sessions	**Pressure stimulation site**: 5 cm away from the left paraspinal process of the fifth lumbar vertebra **Axial T1WI**: FOV: 230 mm × 208 mm × 125 mm - TR = 1999 ms, TE = 10 ms - Flip Angle: 90° - Slice Thickness/Gap: 6 mm/1 mm - voxel size: 0.8 mm × 1 mm **Axial T2WI**: FOV: 230 mm × 208 mm × 125 mm - TR = shortest, TE = 110 ms - Flip Angle: 90° - Slice Thickness/Gap: 6 mm/1 mm - voxel size: 0.76 mm × 0.95 mm **Sagittal T2WI**: FOV: 230 mm × 230 mm × 125 mm - TR = 4,000 ms, TE = 110 ms - Flip Angle: 90° - Slice Thickness/Gap: 6 mm/1 mm **T1WI-3D-TFE sequence**: FOV: 240 mm TR = shortest, TE = shortest Flip angle: 8° Voxel size: 1.1 mm × 1.1 mm × 0.6 mm **BOLD imaging sequence parameters**: TR = 3,000 ms, TE = 50 ms Flip angle: 90° FOV = 240 mm Reconstruction matrix: 144 Number of slices: 32 slices Slice thickness = 4 mm, spacing = 1 mm Pixel size: 3.73 mm × 3.75 mm × 4.0 mm	(1) 4 cases effective: Post-treatment VAS change rate >80%, ODI change rate >45%. Brain activity was mainly inhibited, with the primary areas of inhibition being the right prefrontal cortex (middle frontal gyrus, inferior frontal gyrus) and right cerebellar tonsil (2) 4 cases ineffective: Brain activity was mainly enhanced, with the enhanced areas being the bilateral frontal lobes (superior frontal gyrus, middle frontal gyrus), precentral gyrus, cingulate gyrus, and cerebellum (anterior/posterior lobes)
Cao et al. (2017) (50)	Lumbar disc herniation Inclusion criteria: Aged 18–65 years, all experiencing left-sided nerve root radiating pain, had not taken any sedatives within 3 days prior to participation, and reported no discomfort before the trial; MRI-confirmed lumbar disc herniation; VAS ≥ 4 points; Experimental group (acupoint massage): Average age 39.50 ± 5.04; average disease duration 3.40 ± 1.13 months Control group (non-acupoint massage): Average age 38.70 ± 6.34; average disease duration 4.10 ± 1.54 months	Experimental Group: 10 cases, 5 males and 5 females Control Group: 10 cases, 6 males and 4 females	Experimental Group: Acupoints selected: Bladder meridian points (Chengfu, Yinmen, Weizhong, Chengjin, Chengshan), pressure measured by a pressure detector at 5.6 KG, frequency of 60 times/min. Each acupoint was pressed for 3–5 min, totaling 15–20 min per session, once a day for three consecutive days Control Group: Lateral to the Bladder meridian, 3 cun and 2 cm away. Manipulation method was the same as the treatment group	**Pressure stimulating sites**: left waist and left lower limb **T1WI**: Detailed parameters not provided **T2WI**: Detailed parameters not provided **T2-FLAIR**: Detailed parameters not provided **T1WI-3D-TFE sequence**: Detailed parameters not provided	(1) Brain region changes in the experimental group: Average brain signals increased in the right anterior cingulate gyrus, knee of the cingulate gyrus, insula, lentiform nucleus, nucleus accumbens, periaqueductal gray of the midbrain, and hypothalamus; average brain signals decreased in the hippocampus, amygdala, and globus pallidus of the lentiform nucleus (2) Brain region changes in the control group: Average brain signals increased in the right insula, superior temporal gyrus, and nucleus accumbens; average brain signals decreased in the lentiform nucleus, hippocampus, amygdala, and globus pallidus of the lentiform nucleus
Tan et al. (2019) (43)	Lumbar disc herniation, duration of illness >3 months Inclusion criteria: right-handed; age 20–70 years; lumbar disc herniation confirmed by CT/MRI; VAS ≥ 30; modified ODI ≥ 20%; have not recently taken analgesics, neurotrophic drugs or sedatives, and have not undergone systematic treatment LDH group: mean age 38.95 ± 11.49 years Healthy control group: no statistically significant difference with the LDH group	LDH group: 20 cases, 8 males and 12 females Healthy control group: 20 cases, 8 males and 12 females	Spinal manipulation therapy, 20 min, one session	**Routine cranial MRI plain scan (Three-dimensional magnetization prepared rapid acquisition gradient echo sequence, MP-RAGE)**: Repetition time (TR) / Echo time (TE): 2100 ms / 4.38 ms Flip angle: 8° Field of view (FOV): 220 mm Slice thickness: 1 mm Resolution: 0.861 mm × 0.861 mm Scanning orientation: sagittal Number of slices: 160 **BOLD fMRI, echo-planar imaging sequence**: Repetition time (TR) / Echo time (TE): 2500 ms / 30 ms Slice thickness: 3 mm Slice gap: 0.5 mm Field of view (FOV): 256 mm × 256 mm Matrix: 64 × 64 Flip angle: 90° Scanning condition: The subject keeps their eyes closed and rests quietly, without performing any tasks	(1) There were significant differences in VAS scores and modified ODI scores before and after the intervention (2) Brain region changes before and after intervention: increased ALFF in the posterior lobe of the cerebellum; decreased ALFF in the bilateral middle frontal gyri, right cingulate gyrus, and right angular gyrus (3) Compared with HC: differences in the posterior cingulate gyrus, superior frontal gyrus, and left supplementary motor area prior to intervention were not statistically significant; there was a decrease in ALFF value in the right precuneus (4) Correlation analysis: ALFF value of the right middle frontal gyrus was negatively correlated with the change rates of VAS/ODI
Wen et al. (2022) (44)	Lumbar disc herniation Inclusion criteria: Right-handed; age 20–60 years; VAS ≥ 30/100; lumbar disc herniation confirmed by MRI; Chinese version of the Oswestry Disability Index (C-SFODI) ≥ 20%; no recent use of analgesics, neurotrophic drugs, or sedatives, and no recent systematic treatment LDH group: Average age: 32.2 ± 9.5 years Healthy control group: no statistically significant difference compared to LDH group	LDH patient group: 27 cases, 17 males and 10 females Healthy control group: 28 cases, 17 males and 11 females	Spinal manipulation therapy, 3 times per week, a total of 6 sessions (about 2 weeks), each session lasting 25 min	**Resting-state functional images** **were obtained using an gradient echo-planar imaging sequence**: Functional MRI: 43 interleaved axial slices, matrix size = 64 × 64 field of view (FOV) = 220 mm × 220 mm repetition time (TR) = 2,000 ms, echo time (TE) = 30 ms flip angle = 90 degrees slice thickness = 3.2 mm gap = 0 (voxel size 3.4 × 3.4 × 3.2) number of volumes = 230 **Structural MRI parameters, sequence type: Magnetization Prepared Rapid Gradient Echo (MPRAGE, T1-weighted 3D sequence)**: Sequence = SPGR, sagittal slices, slice number = 176 matrix size = 256 × 256 FOV = 256 × 256 mm TR/TE = 8100/3.1 ms flip angle = 8 degrees slice thickness = 1 gap = 0 (isotropic voxel size = 1 × 1 × 1) All participants were asked to keep their eyes closed and not to think about anything and not to fall asleep during scanning	(1) There was a significant difference in VAS and C-SFODI scores before and after intervention (2) Brain region changes in the LDH group before intervention: compared to the healthy control group, in the conventional frequency band (0.01–0.08 Hz), fALFF in the right lingual gyrus (Lingual_R) was increased; in the Slow - 4 frequency band (0.027–0.073 Hz), fALFF in the left cerebellar Crus1 area (Cerebelum_Crus1_L) was increased; no significant difference in the Slow - 5 frequency band (0.01–0.027 Hz) (3) Brain region changes before and after intervention: after intervention, fALFF in the right lingual gyrus significantly decreased, returning to the level of HCs; after treatment, fALFF in the left cerebellar Crus1 area significantly decreased, also returning to the level of HCs (4) Correlation analysis: Changes in ALFF/fALFF were not significantly correlated with improvement in VAS
Zhou et al. (2023) (42)	Lumbar disc herniation Inclusion criteria: Persistent low back pain or radicular leg pain for ≥6 months; ineffective conservative treatment; MRI-confirmed lumbar disc herniation; VAS pain score ≥4; no recent use of analgesics, neurotrophic drugs, or sedatives, and no recent systematic treatment LDH group: Mean age: 41.28 ± 14.68 years Healthy control group: No statistically significant differences compared to the LDH group	LDH group: 21 cases, 11 males, 10 females Healthy control group: 21 cases, 9 males, 12 females	Lever Positioning Manipulation, single treatment session, 30 min	**Functional parameters (echo-planar imaging sequence)**: Number of slices: 36 slices Repetition time (TR): 2,000 ms Echo time (TE): 40 ms Slice thickness: 3.0 mm (no gap) Voxel size: 3.75 mm × 3.75 mm × 4.0 mm Flip angle: 90° Field of view (FOV): 240 mm × 240 mm Data matrix: 64 × 64 Number of scan layers: 40 layers Scan duration: 8 min **Structure-like parameters (three-dimensional turbo fast echo T1WI sequence)**: Number of slices: 176 slices TR/TE: 7.90/3.06 ms Slice thickness: 1.0 mm (no gap) Field of View (FOV): 256 mm × 256 mm Acquisition matrix: 256 × 256 Fluorescein angiography parameter: 10° Scan duration: 6 min	(1) There were significant differences in VAS pain scores and JOA function scores before and after intervention Brain function changes: (2) Pre-intervention changes in the LDH group’s brain regions: Compared to the healthy control group, the ALFF value of the right precuneus increased, while the ALFF value of the left middle orbital frontal gyrus decreased. ReHo in the left middle/superior orbital frontal gyrus decreased, while ReHo in the right middle frontal gyrus increased (3) Comparison before and after intervention: After intervention, ALFF value in the right middle frontal gyrus increased, ALFF value in the left precentral gyrus decreased; ReHo in the right middle frontal gyrus increased, ReHo in the left precentral gyrus decreased (4) Correlation analysis: The ALFF value of the right middle frontal gyrus was positively correlated with the improvement rate of JOA scores, while no significant correlation was found between other brain regions and changes in VAS/JOA
Zhou et al. (2024) (46)	Lumbar disc herniation Inclusion criteria: Age 18–65 years; right-handed; MRI-confirmed lumbar disc herniation; chronic non-acute phase (symptoms >2 weeks), VAS > 4 points, JOA < 15 points; no recent use of analgesics, neurotrophic drugs, or sedatives, and no systematic treatment Treatment group: average age: 33.57 ± 7.31 years; average disease duration: 59.50 ± 11.44 months Placebo group: average age: 34.23 ± 7.44 years; average disease duration: 57.34 ± 10.93 months Healthy control group: average age: 33.68 ± 7.41 years	Treatment group: 28 cases, 15 males, 13 females; Placebo group: 31 cases, 16 males, 15 females; Healthy control group: 28 cases, 14 males, 14 females	The treatment group received spinal manipulation therapy, 30 min per session, every other day, for a total of 14 sessions The placebo group used the MedX 1,100 laser device to simulate the treatment environment, with the same regimen as the treatment group	**T1 Structural Imaging Sequence: Gradient echo sequence 3D-T1W1**: TR/TE: 2530 ms/2.89 ms Flip angle (FA): 9° Number of layers: 249 Layer thickness/Gap:1.20 mm/1.00 mm Voxel size: 1 × 1 × 1 mm^3^ Field of view (FOV): 256 × 256 mm^2^ Matrix size: 64 × 64 Resolution: 1 mm Scan time: 5 min and 53 s **Rs-fMRI sequence: Echo-planar imaging (EPI) sequence**: TR/TE: 2680 ms/30 ms. Number of layers: 43 Layer thickness/Gap: 3.00 mm/1.00 mm Voxel size: 3 × 3 × 3 mm^3^ FOV: 192 × 192 mm^2^ Matrix size: 64 × 64 Scan time: 10 min and 53 s	(1) After treatment, the treatment group showed significant differences in both VAS pain scores and JOA functional scores; in the placebo group, the post-treatment VAS pain score showed significant difference, but there was no significant difference in JOA functional score (2) Before intervention, there were differences between LDH patients and healthy controls in the left precentral gyrus and the left inferior frontal gyrus, opercular part (3) After intervention in the treatment group, significant changes were observed in the right angular gyrus and the left middle temporal gyrus; in the placebo group, significant changes occurred in the right superior parietal gyrus and the left temporoparietal junction, superior division before and after intervention (4) The improvement rates of right ANG were significantly positively correlated with VAS/JOA; whole-brain analysis with the right ANG as the region of interest: enhanced connectivity from right ANG to left middle orbital gyrus, and decreased connectivity from right ANG to right middle frontal gyrus
Xiaomin (2024) (45)	Lumbar disc herniation Inclusion criteria: Age 20–60 years; right-handed; MRI-confirmed lumbar disc herniation; VAS score ≥3, ODI score ≥20; have not recently taken painkillers, neurotrophic drugs, or sedatives, and have not undergone systematic treatment LDH group: average age 32.2 ± 9.5 years Healthy control group: no statistically significant difference compared to the LDH group	LDH group: 27 cases, 17 males, 10 females Healthy control group: 28 cases, 17 males, 10 females	Spinal manipulation therapy treatment, three times per week, for 2 weeks (a total of 6 sessions), 25 min per session	**Function MRI**: 43 intersecting axes slice orientation, matrix size = 64 × 64 FOV = 220 mm × 220 mm, TR = 2,000 ms TE = 30 ms flip angle = 90 degrees slice thickness = 3.2 mm inter-slice interval = 0 (voxel dimensions 3.4 × 3.4 × 3.2, volume number = 230.2) **Structure MRI: Sequential = SPGR**: vector slices, number of slices = 176 matrix size = 256 × 256 FOV = 256 × 256 mm TR/TE = 8100/3.1 ms rotation angle = 8 degrees, slice thickness = 1 interval = 0 (isotropic voxel size = 1 × 1 × 1) during the resting state fMRI scanning period, all participants are required to close their eyes, not think about anything, and not fall asleep	(1) There were significant differences in VAS and JOA scores before and after the intervention (2) Before the intervention, compared with the healthy control group, the left orbital middle frontal gyrus ReHo decreased; there was no difference in static functional connectivity, the variability of dynamic functional connectivity in the left fusiform gyrus decreased, while the variability of dynamic functional connectivity increased in the left orbital inferior frontal gyrus and the left precuneus (3) After the intervention, the ReHo value of the left orbital middle frontal gyrus significantly increased, approaching normal levels, and only the dFC variability in the left fusiform gyrus within the left orbital middle frontal gyrus returned to healthy levels
Zhou et al. (2024) (46)	Lumbar disc herniation Inclusion criteria: Age 18–65; right-handed; lumbar disc herniation confirmed by MRI; SAS score ≥50, SDS score ≥53; no recent use of painkillers, neurotrophic drugs, or sedatives, and no history of systematic treatment LDH group: Mean age 33.75 ± 7.50 Healthy control group: No statistically significant difference from the LDH group	LDH group: 28 cases, 14 males, 14 females Healthy control group: 28 cases, 15 males, 13 females	Lever Positioning Manipulation, 30 min each session, once every other day, 14 sessions over 4 weeks	**T1 structural imaging scan sequence**: gradient echo sequence 3D-T1WI TR/TE: 2,530 ms/2.89 ms Flip angle (FA): 9° Number of slices: 249 Slice thickness/spacing: 1.20 mm/1.00 mm Voxel size: 1 mm × 1 mm × 1 mm Field of view (FOV): 256 mm × 256 mm Matrix size: 64 × 64 Resolution: 1 mm. Scan time: 5 min 53 s **rs-fMRI scanning sequence**: an echo-planar imaging (EPI) sequence TR/TE: 2,680 ms/30 ms Number of slices: 43 Slice thickness/inter-slice gap: 3.00 mm/1.00 mm Voxel size: 3 mm × 3 mm × 3 mm FOV: 192 mm × 192 mm matrix size: 64 × 64 Scanning time: 10 min 53 s	(1) There was a significant difference in SDS and SAS scores before and after intervention (2) Before intervention, compared with the healthy control group, Fisher’s Z functional connectivity (zFC) of the left precentral gyrus and medial/paracingulate gyri (ECN node) was significantly increased (3) In the LDH group, before and after intervention: zFC of the left temporal pole: middle temporal gyrus (DMN node) and orbital part of the superior frontal gyrus (ECN node) were significantly increased (4) Correlation analysis: zFC of the left temporal pole: middle temporal gyrus was strongly positively correlated with SAS/SDS scores
Zhou et al. (2024) (46)	Lumbar disc herniation Inclusion criteria: Age 18–60 years; right-handed; patients with mild to moderate pain and functional impairment in the non-acute phase (onset more than 2 weeks); MRI-confirmed lumbar disc herniation; VAS > 4 points, JOA < 15 points; no recent use of analgesics, neurotrophic drugs, or sedatives, and no recent systematic treatment LDH group: Mean age 37.47 ± 12.97 years Healthy control group: No statistically significant differences compared to the LDH group	LDH group: 19 cases, 10 males, 9 females Healthy control group: 20 cases, 10 males, 10 females	Leverage Positioning Manipulation, single session, 30 min	**T1 structural imaging was performed with a gradient echo sequence, 3D-T1W1**: TR/TE: 7.90 ms/3.06 ms The flip angle (FA): 15° Number of slices: 249 Slice thickness: 1.2 mm interslice gap: 1 mm FOV: 240 × 240 mm^2^ Matrix size: 512 × 512 Resolution: 1 mm The scan covered the entire brain **The rs-fMRI scan sequence used an echo-planar imaging (EPI) sequence**: TR/TE: 2,000 ms/40 ms The scan included 40 slices with a slice thickness of 3.0 mm Voxel size: 3.75 × 3.75 × 3 mm FOV: 240 × 240 mm^2^ matrix size: 64 × 64 Scan duration was 8 min	(1) There were significant differences in VAS and JOA scores before and after the intervention (2) Before the intervention, compared with the healthy control group, the mALFF values increased in the left superior parietal gyrus, bilateral precuneus, and right dorsolateral superior frontal gyrus, while the mALFF value decreased in the left postcentral gyrus; the right precuneus showed the most significant difference (3) After the intervention, functional connectivity was enhanced in brain regions such as the left medial and paracingulate gyri, left precuneus, and right posterior cingulate gyrus
Zhou et al. (2025) (47)	Lumbar disc herniation Inclusion criteria: MRI-confirmed lumbar disc herniation, neurological localization consistent with imaging findings; age 18–65 years; right-handed; chronic, non-acute phase (symptoms >2 weeks); VAS > 4, JOA < 15; no use of painkillers/neurotrophic drugs/sedatives, no systematic treatment or manual therapy within the past month LDH group: average age 34.06 ± 7.27 years Healthy control group: no statistical difference compared to LDH group	LDH group: 31 cases, 17 males, 14 females Healthy control group: 28 cases, 14 males, 14 females	Spinal manipulation therapy, once every other day, for a total of 14 sessions	**T1 structural imaging sequence parameters**: Sequence Type: Gradient echo sequence 3D-T1W1 TR/TE: 2530 ms / 2.89 ms Flip angle (FA): 9° Number of slices: 249 slices Slice thickness/spacing: 1.20 mm / 1.00 mm Voxel size: 1 mm × 1 mm × 1 mm Field of View (FOV): 256 mm × 256 mm Matrix size: 64 × 64 Resolution: 1 mm Scan time: 5 min 53 s **Resting-state functional MRI sequence parameters**: Sequence type: Echo-planar imaging (EPI) TR/TE: 2680 ms / 30 ms Number of slices: 43 slices Slice thickness/gap: 3.00 mm / 1.00 mm Voxel size: 3 mm × 3 mm × 3 mm Field of view (FOV): 192 mm × 192 mm Matrix size: 64 × 64 Scan time: 10 min 53 s	(1) There were significant differences in VAS and JOA scores before and after the intervention (2) After the intervention, ALFF values in the right superior temporal gyrus increased, ALFF values in the left paracentral lobule decreased, ReHo in the right olfactory cortex increased, and ReHo in the right cuneus decreased (3) Before the intervention, compared with the healthy control group, ALFF in the left temporal pole of the superior temporal gyrus increased, ALFF in the left middle frontal gyrus decreased, ReHo in the left postcentral gyrus increased, and ReHo in the left parahippocampal gyrus decreased (4) Correlation analysis: ReHo value in the right cuneus was strongly positively correlated with VAS improvement rate and positively correlated with JOA improvement rate

### 3.2 Population characteristics and fMRI data collection

Among the studies included, all of which concentrated on patients with LDH, there were notable differences in the inclusion criteria that merit a systematic description. Specifically, concerning the requirement for disease duration, two studies (41, 42) mandated that patients have experienced the condition for a minimum of 6 months, one study (43) required a duration exceeding 3 months, while other studies did not explicitly specify a minimum duration but indicated that patients should be in a non-acute phase of the disease. Regarding demographic characteristics, although there were minor variations in age and sex composition across the studies, the patient groups were predominantly homogeneous, comprising only chronic LDH patients who were right-handed and had not recently used related medications. These characteristics provide a representative profile of the basic population composition used in current clinical research in this field.

Notably, various studies exhibit significant discrepancies in the data acquisition parameters for functional magnetic resonance imaging. Such methodological variations are crucial for the comprehensive interpretation of the research findings. Yuan et al. (41) established the repetition time (TR) of the BOLD sequence at 3,000 ms, which is considerably higher than that in other studies. The duration of the TR directly influences the capability of interslice coverage and temporal resolution: a longer TR facilitates broader whole-brain coverage but diminishes temporal resolution, thereby affecting the ability to capture rapid neural dynamic processes and increasing the risk of motion artifacts.

Furthermore, four studies (41, 42, 44, 45) employed anisotropic voxels as spatial acquisition parameters, indicating that the voxel sizes varied across the X, Y, and Z dimensions. For instance, Zhou et al. (42) utilized functional voxels measuring 3.75 mm × 3.75 mm × 4.0 mm in size. Such configurations are typically selected to optimize the balance between the scanning efficiency and signal-to-noise ratio. However, they may also lead to inconsistent spatial resolutions across different directions, thereby impacting the precise spatial localization of brain activation signals. Given the diversity of research objectives and experimental designs, imaging parameters exhibit significant variability across studies. This variability underscores the absence of a standardized image acquisition protocol in current research. Consequently, when synthesizing existing evidence in this field, it is crucial to consider the impact of these methodological differences and avoid directly comparing quantitative results from different studies.

### 3.3 Basic characteristics of interventions

Six studies used standard spinal manipulation techniques (S-SMT) (41, 43–47). The specific procedure was as follows: the patient first lay in the prone position to receive soft tissue relaxation techniques on the lumbar region, and then shifted to the lateral position, facing the clinician with the affected side upwards. The clinician passively flexed the participants’ hip and knee joints to induce lumbar flexion until movement was detected at the spinous process of the affected lumbar vertebra. Next, the clinician passively rotated the participant’s trunk away from the affected side until rotation was felt at the vertebra above the suspected lesion. The clinician then applied a quick simultaneous thrust to the participant’s shoulder (posterior force) and pelvis (anterior force), generating a rotational moment at the target vertebral segment. If cavitation (i.e., an audible pop) was produced, the treatment was considered complete. If no cavitation occurred, the participant was repositioned, and the procedure was reattempted, with a maximum of two attempts per side. If no cavitation was elicited after four attempts (two per side), the treatment was considered complete.

Three studies used lever-positioning manipulation (LPM) (42, 48, 49). The specific procedure was as follows: the patient was placed in the prone position and received soft tissue relaxation techniques in the lumbar region. The protruding lumbar intervertebral disc served as the positioning point, and the patient was asked to flex their knees and hips and cross their lower limbs. The clinician used the right elbow’s olecranon to act on the patient’s protruding disc, grasped the patient’s ankles with both hands, and induced hyperflexion and anterior flexion of the lumbar spine by utilizing the lever arm. The lumbar area was then quickly lifted upward and backward, and when resistance was encountered, a rapid adjustment was performed. During manipulation, the patient was instructed to exhale, and at the end, inhale, followed by a quick controlled 5° adjustment. Breath-holding should be strictly avoided. Finally, the patient was instructed to rest in the supine position on the bed for 20 min.

One study used traditional Chinese medicine acupoint massage techniques (50). The specific procedure was as follows: the physician used the tip of the right thumb to apply pressure to the selected acuppoint, with the wrist relaxed, shoulder down, elbow dropped, and the wrist suspended. The elbow was positioned slightly below the wrist to apply a vertical force without causing lateral movement. The force, measured using a pressure detector, was 5.6 kg at a frequency of 60 times per minute. Each acupoint was pressed for 3–5 min, with a total treatment time of 15–20 min.

### 3.4 Reported changes in functional connectivity

#### 3.4.1 Immediate effects of spinal manipulation

In the study conducted by Wenli et al. (43), a single S-SMT intervention was administered to patients with LDH. Clinical symptoms were evaluated using the Visual Analog Scale (VAS) and Oswestry Disability Index (ODI). fMRI analysis performed immediately post-intervention revealed a significant increase in the amplitude of low-frequency fluctuations (ALFF) in the posterior cerebellar lobe of patients with LDH. Conversely, the ALFF values in the bilateral middle frontal gyrus (MFG), right cingulate gyrus, and right angular gyrus exhibited a significant decrease. Furthermore, the study identified a negative correlation between changes in ALFF in the right middle frontal gyrus (Frontal_Mid_R) and the improvement rates of the VAS/ODI scores.

In a separate investigation by Zhou et al. (42), patients with LDH underwent a single session of lever position manipulation. The evaluation was conducted using the VAS and Japanese Orthopedic Association (JOA) scores. The fMRI results obtained within 1 h post-intervention indicated a significant enhancement in ALFF values and regional homogeneity (ReHo) in the right middle frontal gyrus (Frontal_Mid_R), whereas a significant reduction was observed in the left precentral gyrus. Notably, the study found a positive correlation between the enhancement of ALFF in the right middle frontal gyrus (Frontal_Mid_R) and the improvement rate of the JOA score, whereas no significant correlation was observed between other brain regions and changes in VAS/JOA scores.

In another study, Xing-chen et al. (49) reported alterations in functional connectivity (FC) in patients with LDH following a single session of lever position manipulation. The FC among brain regions, including the left medial and paracingulate gyri, left precuneus, and right posterior cingulate cortex, was significantly increased.

#### 3.4.2 Long-term effects of spinal manipulation

Yuan et al. (41) administered S-SMT to eight patients diagnosed with lumbar disc herniation and conducted six sessions over an 18-day period. Of these patients, four demonstrated effective outcomes, as indicated by a post-treatment VAS change rate exceeding 80% and ODI change rate surpassing 45%. The study revealed that patients who responded effectively to treatment exhibited an increased local pain threshold in the lumbar region compared to pre-treatment levels. Additionally, their brain functional activity was predominantly characterized by inhibition of the prefrontal cortex and cerebellum. Conversely, patients who did not respond to treatment showed no change in their local lumbar pain threshold, and their brain functional activity was primarily characterized by heightened activity in the bilateral frontal lobes, cerebellum, precentral gyrus, cingulate gyrus, and other regions.

Zhou et al. (46) implemented an S-SMT protocol for patients with LDH, involving treatments every other day for 14 sessions, with a placebo control group. The placebo group used the MedX 1,100 laser device to simulate the treatment environment, with the same course as that of the treatment group. Researchers used the VAS and JOA for evaluations. The results showed significant differences in both VAS pain scores and JOA function scores in the treatment group after the intervention, whereas in the placebo group, only the VAS pain score showed a significant difference after the intervention. Compared to healthy adults, patients had increased activation in the left precentral gyrus (PreCG) and left inferior frontal gyrus, pars orbitalis (IFGoperc). After 14 intervention sessions, patients exhibited significant changes in the activity of the right angular gyrus (ANG) and left middle temporal gyrus (MTG), whereas in the placebo group, significant changes appeared in the right superior parietal gyrus and left temporoparietal junction. Further analysis using the right ANG as a region of interest (ROI) showed enhanced connectivity from the right ANG to the left middle orbital frontal gyrus (ORBmid), whereas connectivity from the right ANG to the right middle frontal gyrus (MFG) decreased. Correlation analysis revealed that changes in right ANG activity were significantly positively correlated with improvements in the VAS and JOA scores.

Xingchen et al. (48) also applied an S-SMT regimen to patients with LDH, with treatments every other day for a total of 14 sessions, and used the VAS and JOA for evaluation. The results again showed significant differences in both VAS pain and JOA function scores after treatment in the intervention group. Compared with healthy adults, patients with LDH exhibited increased ALFF in the left superior temporal pole, decreased ALFF in the left middle frontal gyrus (MFG_L), increased ReHo in the left postcentral gyrus, and decreased ReHo in the left parahippocampal gyrus. After 14 SMT sessions, patients with LDH showed increased ALFF values in the right superior temporal gyrus, decreased ALFF in the left paracentral lobule, increased ReHo in the right olfactory cortex, and decreased ReHo in the right cuneus. Correlation analysis indicated that ReHo values in the right cuneus were strongly positively correlated with the improvement rate of the VAS and positively correlated with the improvement rate of the JOA.

Zhou et al. (47) further adopted a LPM for patients with LDH, with treatments every other day for a total of 14 sessions. Researchers used the Self-Rating Anxiety Scale (SAS) and Self-Rating Depression Scale (SDS) for the evaluation. The results showed significant differences in both the SAS and SDS scores after the intervention in the treatment group. fMRI results indicated that Compared to healthy adults, LDH patients exhibited significantly enhanced Fisher’s Z functional connectivity (zFC) in the left precentral gyrus and medial/paracingulate gyri (ECN nodes). After intervention, patients with LDH had significantly enhanced zFC in the left temporal pole, middle temporal gyrus (DMN nodes), and orbital superior frontal gyrus (ECN nodes). Correlation analysis revealed a strong positive correlation between zFC in the left temporal pole, middle temporal gyrus, and SAS/SDS scores. Two studies adopted identical intervention frequencies and protocols (46, 48), and their results collectively highlighted the key role of normalization of the parietal-occipital cortex function in SMT’s therapeutic effect of SMT on pain symptoms. Another study reported that leverage positioning manipulation plays a role in relieving anxiety and depression during the treatment of lumbar disc herniation and that enhanced connectivity between the temporal pole and middle temporal gyrus was related to improvements in anxiety and depression (47).

Wen et al. (44) provided S-SMT intervention for patients with LDH three times a week for a total of six sessions. Clinical symptoms were assessed using the VAS and the Chinese version of the Oswestry Disability Index (C-SFODI). The results showed significant changes in both the VAS and C-SFODI scores after the intervention. The fMRI results revealed that, compared to healthy controls, patients with LDH who did not receive intervention demonstrated increased fALFF in the right lingual gyrus (Lingual_R) in the conventional frequency band (0.01–0.08 Hz) at baseline, and increased fALFF in the left cerebellum Crus1 region (Cerebelum_Crus1_L) in the Slow-4 band (0.027–0.073 Hz), but no significant difference in the Slow-5 band (0.01–0.027 Hz). After intervention, the fALFF values of patients with LDH in the right lingual gyrus and left cerebellum Crus1 region significantly decreased to levels comparable to those of healthy controls. However, correlation analysis indicated that changes in fALFF in the above brain regions were not significantly correlated with improvements in VAS scores.

Xiaomin et al. (45) applied a S-SMT protocol to patients with LDH, using three sessions per week for a total of six sessions. Researchers used the VAS and ODI for clinical evaluation. The results indicated significant differences in both the VAS and JOA scores after the intervention in the treatment group. fMRI results demonstrated that Compared to healthy controls, untreated LDH patients at baseline showed decreased ReHo in the left orbital middle frontal gyrus (LO-MFG), with no difference in static functional connectivity brain regions, decreased variance in dynamic functional connectivity in the left fusiform gyrus, and increased dynamic functional connectivity variance in the left orbital inferior frontal gyrus and the left precuneus. After completing the intervention, ReHo values in the left orbital middle frontal gyrus (LO-MFG) significantly increased and approached normal levels, with only the left fusiform gyrus dFC variance in the LO-MFG recovering to healthy levels.

#### 3.4.3 Acupoint specificity

Cao et al. (50) conducted a study investigating the effects of Traditional Chinese Medicine (TCM)-based spinal manipulation on brain function in patients with LDH. Using a randomized controlled design, participants were allocated to either an intervention or a control group. The intervention group received targeted pressure application on specific acupoints of the Bladder Meridian (BL), namely Chengfu (BL36), Yinmen (BL37), Weizhong (BL40), Chengjin (BL56), and Chengshan (BL57). The control group received equivalent pressure applied to non-acupoint, non-meridian sites located 3 cm and 2 cm lateral and perpendicular to the Bladder Meridian trajectory. The fMRI analysis demonstrated that the intervention group (acupoint pressure) exhibited significant alterations in brain activation patterns, with particularly prominent activation observed in the Anterior Cingulate Cortex (ACC) and Periaqueductal Gray (PAG). In contrast, the control group (non-acupoint pressure) showed a significantly greater reduction in signal intensity than the intervention group within the Putamen, Hippocampus, Amygdala, and Globus Pallidus. Notably, post-intervention analysis revealed diametrically opposed changes in putamen signal intensity between the groups: the intervention group exhibited increased signal intensity, whereas the control group demonstrated decreased signal intensity.

#### 3.4.4 Functional connectivity changes and clinical relevance

Two studies reported a correlation between brain changes in the parietal-occipital lobe region and improvements in the VAS and JOA scores (46, 47). One study showed that after SMT, changes in right ANG activity were positively correlated with improvements in the VAS and JOA scores (46). Another study indicated that after SMT, the ReHo value in the right cuneus was strongly positively correlated with the VAS improvement rate and positively correlated with the JOA improvement rate (47). Additionally, two studies reported a correlation between brain changes in the prefrontal cortex and VAS and JOA scores (42, 43). After a single S-SMT session, changes in the right middle frontal gyrus ALFF value were correlated with changes in the VAS/ODI (43), and following a single lever position manipulation, the ALFF value of the right middle frontal gyrus (Frontal_Mid_R) was positively correlated with the improvement rate in JOA scores but had no correlation with changes in VAS (42). Moreover, after multiple sessions of lever position manipulation, a strong positive correlation was found between the zFC in the left middle temporal gyrus and SAS/SDS scores (48). However, in the study by Wen et al., no correlation was found between ALFF/fALFF changes in brain regions and the VAS score (44).

In addition, patients with LDH exhibited significantly increased abnormal brain activity in the right pre-wedge frontal lobe ALFF (42, 49) and enhanced abnormal activity in the left precentral gyrus (46, 48) at baseline. Cerebellar activity modulation was observed after both single and multiple SMT sessions increased posterior lobe ALFF (43) and decreased fALFF in the cerebellar Crus1 area (44).

## 4 Discussion

Although numerous studies have employed fMRI to investigate the effects of manual therapy on brain function in patients with LDH, these studies often fail to clearly report fMRI scanning parameters, preprocessing procedures, and calculation methods for indicators. They also did not systematically control for confounding factors, such as consistency in disease duration or other analgesic treatments. Most studies lack quantification of manual therapy parameters or placebo-controlled designs, do not provide baseline brain activity data to match LDH groups with healthy controls, and do not verify the correlation between changes in brain activity and improvements in clinical pain or dysfunction. Additionally, the single-session intervention designs of some studies make it difficult to reflect the true therapeutic effects.

Spinal manipulation can be divided into thrust and non-thrust techniques. The main differences between thrust manipulation and non-thrust manipulation/mobilization lie in the speed and amplitude of the applied force, as well as whether a “cracking” sound (cavitation) is produced. Thrust manipulation is characterized by a high velocity and low amplitude. Among the three manipulation modes in this study, acupoint massage belongs to non-thrust manipulation, while S-SMT and LPM, although partly non-thrust, still have key components that fall under the thrust manipulation. Previous studies (51) have found that when both techniques are applied to patients with lumbar-related lower back pain, there are no differences between the groups in terms of FABQ-W and ODI scores, as well as self-reported questionnaires, including the Numerical Pain Rating Scale (NPRS). Additionally, there was no interaction effect between time and group.

This study is the first to systematically demonstrate that spinal manipulation alleviates pain and emotional disorders by regulating the key brain regions. The included studies found that the following may be the key brain areas through which spinal manipulation techniques modulate pain perception and cognitive processing in patients with LDH: right MFG, right ANG, right cuneus, left MTG, and LO-MFG.

### 4.1 Differences between SMT and placebo effects in LDH

Zhou et al. (46) conducted a 14-session S-SMT intervention for LDH patients and established a placebo control group; the placebo group received sham laser stimulation, with treatment sessions, environmental simulation, and other aspects consistent with the intervention group. This study provides high-quality evidence for exploring the specific effects of SMT. By comparing its results with those of non-controlled SMT studies, the core clues to the specificity of SMT effects can be clearly revealed.

From the perspective of treatment expectations, the core driving factor of placebo analgesia is the patient’s “analgesic expectation,” a conclusion confirmed by previous research (52): patients’ expectations regarding the efficacy of SMT can directly influence their perception of pain. For example, among healthy volunteers, negative expectations can exacerbate local pain after SMT intervention, while positive expectations may enhance the analgesic effect to some extent. However, the fundamental analgesic effect of SMT does not depend on positive expectations, and its analgesic action has specificity that is independent of psychological expectations.

In terms of clinical outcomes, a placebo-controlled study by Zhou et al. (46) demonstrated that the S-SMT treatment group experienced significant improvements in both the VAS and JOA scores. Conversely, the placebo group showed only a notable reduction in VAS scores, with no meaningful change in JOA scores. This finding corroborates previous research: placebo interventions can only slightly reduce patients’ subjective pain scores and do not enhance lumbar physical function (53, 54). In non-controlled SMT studies, where no placebo group was included, all intervention groups generally reported concurrent improvements in both pain and functional scores. This complicates the task of distinguishing the specific effects of SMT from placebo effects, often resulting in an overestimation of SMT’s role in functional improvement.

From the perspective of brain function activation specificity, patients’ analgesic expectations can activate brain regions such as the left anterior cingulate cortex, right precentral gyrus, lateral prefrontal cortex, and left periaqueductal gray (20, 55). Additionally, the study by Zhou et al. (46) demonstrated that the S-SMT treatment group not only showed specific activation of placebo-expectation-related brain regions, such as the ACC and PAG, but also modulated key nodes of the default mode network, specifically the right angular gyrus and left middle temporal gyrus. Furthermore, changes in activity in the right angular gyrus were significantly positively correlated with improvements in the VAS pain and JOA functional scores. These findings suggest that the analgesic and functional improvement effects of SMT are not merely due to placebo effects mediated by “analgesic expectation.” Instead, based on this mechanism, SMT triggers specific neural regulation through mechanical stimulation, ultimately achieving pain relief and restoration of physical function. This is the core feature that distinguishes SMT from simple placebo.

### 4.2 Analgesic mechanisms of thrust manipulation techniques

Thrust manipulation involves applying a low-speed, continuous, and gentle mechanical force to the skin. This technique can enhance blood circulation by compressing local tissues, thereby reducing the concentration of pain-inducing substances and decreasing their duration of action on pain receptors. Concurrently, massage can accelerate metabolism, supplying more nutrients to injured tissues and expediting the repair process. Furthermore, manipulation techniques can modify abnormal pathological conditions and potentially eliminate the pathogenic factors. The posterior horn of the spinal cord serves as a critical relay station in the analgesic process of massage, underscoring its essential role in integrating and regulating this information. Beyond the spinal cord, the periaqueductal gray matter in the midbrain and the nucleus raphe magnus in the medulla not only constitute the descending pain inhibition system with the spinal cord but also function as signal transduction centers for impulses transmitted from higher centers.

In the thrust manipulation intervention examined in this study, the location of thrust application influenced patient outcomes, with those receiving force at specific acupoints exhibiting activation in regions such as the periaqueductal gray matter of the midbrain and anterior cingulate cortex. The periaqueductal gray matter is a pivotal structure in the endogenous pain modulation system, playing a central role and serving as a crucial brain area for the analgesic effect produced by the activation of higher brain centers. Consequently, thrust manipulation at specific acupoints may activate endogenous pain modulation pathways, inhibit the transmission of pain signals, and thereby achieve an analgesic effect by stimulating increased brain signals and expanding PAG activation.

### 4.3 Analgesic mechanisms of non-thrust manipulation techniques

#### 4.3.1 Analgesic mechanisms mediated by the prefrontal cortex

The right MFG and LO-MFG area together form part of the prefrontal cortex (PFC). As a higher-level integration hub for contextual memory, spatial orientation, event processing, and adaptive responses, the PFC participates in the recognition, transmission, and processing of pain sensations by integrating input and output information and modulating the perception of the spatiotemporal attributes of pain (56). In the studies included in this review, Zhou et al. (42), Wenli et al. (43), and Xiaomin (45) specifically pointed out that there were significant changes in PFC activity in patients after the intervention. Notably, the studies by Zhou et al. (42) and Wenli et al. (43) involved only a single treatment session, yet both found that changes in PFC activity were significantly correlated with VAS scores. The excitability of the PFC is highly correlated with pain response. fMRI evidence indicates that during pain states, PFC excitability increases, while its functional connectivity with the bilateral nucleus accumbens (NAc) decreases. Moreover, using low-frequency repetitive transcranial magnetic stimulation to reduce PFC excitability can exacerbate the expression of pain (57).

It is noteworthy that Wenli et al. (43) observed a decrease in ALFF in the right middle frontal gyrus after intervention, while Zhou’s study showed an increase in ALFF in the same region; yet both interventions produced analgesic effects. At present, the underlying mechanisms behind this contradictory finding have yet to be clarified, and existing studies cannot rule out the confounding effects of factors such as sample size, intervention methods, and individual differences in brain function. Currently, there is a hypothesis that may explain this contradiction, but it still requires further confirmation through high-quality research: the PFC is not a functionally homogeneous brain region, and its different subregions may exert distinct regulatory effects on pain through specific neurotransmitter pathways. For example, noradrenergic neurons in the locus coeruleus (LC), especially its dorsal part, project to the PFC and enhance the excitability of PFC pyramidal neurons by activating α-receptors, thereby inducing pain sensitization (58). In addition, glutamatergic projections from the basolateral amygdala (BLA) to the PFC can enhance pain-related negative emotions, whereas inhibition of GABAergic interneurons within the PFC can exacerbate pain perception (59, 60). Thus, the pathways from the locus coeruleus to the prefrontal cortex (LC-PFC) and from the basolateral amygdala to the prefrontal cortex (BLA-PFC) together create a circuit that promotes pain perception within the prefrontal cortex. Based on this, the SMT used by Wenli et al. (43) may lead to a decrease in ALFF in the right middle frontal gyrus by inhibiting the above-mentioned pro-nociceptive pathways. The reduction in ALFF may reflect the enhanced activity of GABAergic interneurons, which in turn inhibits the excessive excitation of PFC pyramidal neurons, ultimately relieving pain. This also explains the negative correlation found in their study between the degree of ALFF reduction in the right MFG and the improvement rates of the VAS/ODI.

Meanwhile, there are analgesic pathways within the PFC. For example, activation of dopaminergic neurons projecting from the ventral tegmental area (VTA) of the midbrain to the PFC, especially to the prelimbic (PrL) subregion of the medial prefrontal cortex (mPFC), can significantly raise the pain threshold (61, 62). Additionally, activation of glutamatergic projections from the mPFC PrL region to the nucleus accumbens (NAc) by stimulating D1-type dopamine receptor-expressing medium spiny neurons (D1-MSNs) can also mediate analgesic effects (63, 64). Based on this, the following hypotheses are proposed: the lever positioning approach used by Zhou et al. (42) may activate such analgesic pathways, leading to increased ALFF in the right MFG. The rise in ALFF may reflect the enhanced activity of glutamatergic pyramidal neurons, which, in turn, can reduce the transmission of pain signals in the spinal dorsal horn through descending inhibitory pathways. This is consistent with the findings of their study that elevated ALFF in the right MFG was positively correlated with improvement rates in the JOA scores. However, it must be noted that the aforementioned speculations regarding the mechanisms of pain-promoting and pain-relieving pathways are theoretical hypotheses, and their validity requires further verification through large-sample, standardized experimental studies.

#### 4.3.2 Analgesic mechanisms mediated by the occipital cortex

The right cuneus and the right lingual gyrus are both part of the occipital cortex. The right cuneus is primarily involved in visual information processing but is also associated with visuospatial attention, visual imagery, and pain control. In one study included in this review (47), patients showed a significant decrease in ReHo in the right cuneus after 14 sessions of S-SMT, and the ReHo value in the right cuneus was strongly positively correlated with the change rate of the VAS score. The right cuneus, an important component of the visual network (VN), is a key brain region responsible for performing advanced visual perception tasks. Studies have shown that shifting attention using visual cues can effectively increase the pain threshold (65), and this strategy has also been proven effective in clinical settings (66). Related research has found that VN features based on changes in resting-state functional connectivity can distinguish patients with LDH from HCs with an accuracy of 79.3% (67). In addition, gray matter density features in the VN regions can distinguish patients with LDH from HCs with an accuracy of 76% (68).

The right lingual gyrus also participates in early visual information processing, similar to that of the right cuneus. Interactions between the visual cortex and sensory cortex may influence the spatial allocation of attention to pain. Wen et al. (44) found that after S-SMT intervention, the fALFF in the right lingual gyrus significantly decreased, but there was no significant correlation with VAS improvement. Both the right cuneus and right lingual gyrus, which are part of the occipital lobe visual cortex, showed reduced activity after S-SMT treatment; however, only the change in activity of the cuneus was significantly associated with pain improvement. This phenomenon may be due to the fact that the cuneus, as a key node of both the higher-order visual network and the dorsal attention network, has closer connections with brain regions such as the anterior cingulate gyrus, prefrontal cortex, and parietal lobe, which are involved in cognitive regulation and descending pain inhibition pathways. Such connections enable the brain to modulate pain signals more effectively. In contrast, the lingual gyrus is mainly connected to the primary visual area and other regions involved in processing object features in the ventral visual pathway. Therefore, within the visual network of the occipital lobe visual cortex, the right cuneus-centered VN may be the key central network underlying the analgesic effect of S-SMT in patients with LDH. Therefore, the VN centered on the right cuneus may be the key central network through which S-SMT exerts its analgesic effect on patients with LDH.

#### 4.3.3 Analgesic mechanisms mediated by the default mode network

The default mode network (DMN), a core functional network of the brain that is highly active during rest, is closely linked to advanced cognitive functions such as introspection, self-referential thinking, and contextual memory retrieval (69). In chronic pain states, the DMN often exhibits abnormal functional enhancement, and this excessive activation is believed to be associated with the chronicization of pain and accompanying negative emotions. The bilateral angular gyrus, temporal cortex, and precuneus are key nodes of the DMN. The studies included in this review found (42, 46, 49) heightened baseline DMN activity; patients with LDH exhibited increased DMN node activity at baseline, consistent with previous findings of DMN overactivation in chronic pain states. After the S-SMT cycle, changes in the activity of the right angular gyrus were significantly positively correlated with the rates of improvement in patients’ pain intensity and lumbar function scores (46). Further analysis revealed that, following S-SMT, the functional connectivity (FC) between the right ANG and the left middle orbital gyrus (left middle orbital gyrus, ORBmid) was significantly enhanced, whereas the FC with the right MFG was reduced (46). This specific pattern of functional connectivity reorganization suggests that the mechanism by which SMT relieves pain may partly stem from its inhibition or functional re-integration of DMN overactivity.

Another study observed that after treatment with LPM, the fzFC of the left temporal pole: middle temporal gyrus was significantly enhanced, and this enhancement showed a strong positive correlation with improvements in anxiety and depression scores after treatment (48). Both the temporal pole and middle temporal gyrus are important nodes of the DMN, suggesting that LPM may improve emotional comorbidities in patients with LDH by enhancing internal connectivity within the DMN. As a core node of the DMN, the precuneus is deeply involved in advanced functions such as spatial perception, self-referential cognition, and episodic memory (43, 49). Variability in functional connectivity under pain conditions may reflect the brain’s adaptive adjustment mechanisms to maintain specific cognitive and emotional functions.

The mechanism of S-SMT for LDH is not limited to local mechanical adjustments and biomechanical improvements; more importantly, it may regulate pain perception and processing at the central nervous system level by reshaping the functional activity of the brain’s DMN. These multimodal neuroimaging findings, based on rs-fMRI and graph theory attribute analysis, provide a solid network neuroscience foundation for the “brain-bone interaction” theory.

## 5 Strengths and limitations of this scoping review

This review synthesizes research on the brain’s functional mechanisms of SMT in the treatment of LDH. Studies incorporating pharmaceutical interventions or other physical therapies, such as acupuncture, and those lacking fMRI results were excluded. While this screening approach may omit some potential insights into the mechanisms of discogenic pain, our primary objective was to elucidate the brain functional changes induced by SMT in patients with LDH by concentrating on key evidence. Currently, research on the impact of spinal manipulation therapy on brain changes in patients with LDH remains limited, potentially due to the high cost of fMRI testing and the feasibility of subjects undergoing fMRI scans. Most existing studies continue to focus on muscle dynamics and inflammatory substances. However, as research into the correlation between lumbar discogenic pain and changes in brain regions advances, and given that spinal manipulation therapy is the principal conservative treatment for LDH, further research is necessary to explore how spinal manipulation influences changes in brain regions in patients with LDH.

In addition, the small sample size included in the study and the high methodological heterogeneity, which leads to risks of bias and insufficient statistical power, are important factors that limit the conclusions of this research. Although previous studies have pointed out that this issue stems from a lack of funding in SMT mechanism research (70), resulting in limited access to resources and the application of methodological expertise, this limitation still requires special attention. In terms of sample size, the studies included generally had small sample sizes. For example, in this study, Yuan et al. (41) included only 8 patients with LDH, and Cao et al. (50) study on acupoint massage included only 10 patients. Numerous studies have identified small sample size as a significant limitation in neuroscience research (71, 72). Small sample sizes, on one hand, hinder the effective detection of true associations between changes in brain function and clinical indicators, thereby increasing the likelihood of false negatives. In contrast, group differences observed in small-sample conditions are susceptible to the influence of outliers or individual heterogeneity, which not only elevates the risk of false-positive associations but also compromises the stability and reproducibility of research findings. At the methodological level, this study faced significant issues of heterogeneity. First, there is an inconsistency in the fMRI acquisition parameters. For example, Yuan et al. (41) set the TR of the BOLD sequence to 3,000 ms, which is much higher than that in the other included studies. Second, there is a lack of unified standards for intervention protocols, with some studies focusing on the immediate effects of a single SMT intervention, while others analyze the long-term effects of multiple interventions.

This study consolidates previous relevant research and examines the changes in brain regions and analgesic patterns in patients with LDH treated with various thrust-mode spinal manipulative therapy protocols. However, owing to the small sample sizes and methodological heterogeneity in some current studies, findings on the clinical mechanisms of SMT at this stage are only exploratory and should be interpreted with caution.

## 6 Conclusion

While this study systematically demonstrates the potential of spinal manipulation in regulating key brain regions to alleviate pain and emotional disorders, it acknowledges limitations such as the small number of studies and heterogeneity in manipulation techniques. These factors may affect the generalizability of our findings. Future research should aim to address these limitations. The current findings indicate that S-SMT may relieve LDH pain and emotional disorders by regulating the prefrontal cortex, occipital visual network, default mode network, and other brain regions.

### 6.1 Prospects of future study

Future research should first expand the sample size and enhance the representativeness of the participants to ensure that the study population comprehensively reflects the clinical characteristics of patients with LDH. Simultaneously, it is necessary to distinguish between nociceptive and neuropathic pain subtypes to lay the foundation for precise mechanistic exploration. Studies should prioritize standardized designs: matching the course of the disease and prior treatment history among participants, paying attention to the design of placebo control groups to investigate the nonspecific mechanisms behind SMT, and quantifying the biomechanical parameters of the manipulations to ensure repeatability and comparability of interventions. In the data analysis phase, it is important to rigorously adjust for potential confounding factors to reduce research bias. In addition, studies should integrate multi-omics technologies, for example, performing spatial transcriptome sequencing on surgically obtained intervertebral disc tissue to locate regions expressing pain-related neuropeptides and correlate these with changes in brain networks observed via fMRI. Through this multidimensional research approach, we can analyze the brain mechanisms of lumbar disc herniation more comprehensively and in greater depth.

## Data Availability

The original contributions presented in the study are included in the article/Supplementary material, further inquiries can be directed to the corresponding author.

## Glossary

ACC: Anterior cingulate cortex
ALFF: Amplitude of low-frequency fluctuations
ANG: Angular gyrus
BLA: Basolateral amygdala
BOLD: blood oxygen level-dependent
CNS: Central nervous system
CLBP: Chronic low back pain
C-SFODI: Chinese version of the Oswestry disability index
dFC: Dynamic functional connectivity
DMN: Default mode network
FC: Functional connectivity
fMRI: Functional magnetic resonance imaging
fALFF: fractional amplitude of low-frequency fluctuations
JOA: Japanese orthopedic association scores
LC: Locus coeruleus
LDH: Lumbar disc herniation
LPM: lever-positioning manipulation
LO-MFG: Left orbital middle frontal gyrus
MFG: Middle frontal gyrus
MTG: Middle temporal gyrus
mPFC: Medial prefrontal cortex
NAc: Nucleus accumbens
ODI: Oswestry disability index
ORBmid: Middle orbital frontal gyrus
PAG: Periaqueductal gray
PRISMA-ScR: Preferred reporting items for systematic reviews and meta-analyses extension for scoping reviews
PrL: Prelimbic
ReHo: Regional homogeneity
rs-fMRI: Resting-state functional magnetic resonance imaging
SAS: Self-rating anxiety scale
SDS: Self-rating depression scale
S-SMT: Standard spinal manipulation techniques
SMT: Spinal manipulation therapy
TR: Repetition time
VAS: Visual analog scale
VN: Visual network
VTA: Ventral tegmental area
zFC: Fisher’s Z functional connectivity
