# Supplementary material for: Brain functional changes following spinal manipulation therapy in patients with lumbar disc herniation and chronic low back pain: a scoping review
Source: Front Neurol. 2026 Jan 6;16:1712320. doi: 10.3389/fneur.2025.1712320 (PMC12815715; doi:10.3389/fneur.2025.1712320)
Supplement: Supplementary file 1 [file Table_1.docx]

OSF pre-registration: https://doi.org/10.17605/OSF.IO/SEPKF

PubMed Search Results Information Table

| **Number** | **Search Expression** | **Number of Results** |
| --- | --- | --- |
| #1 | (( "Intervertebral Disc Displacement"[Mesh] OR "Intervertebral Disc Displacement"[Mesh] )) OR ( "Intervertebral Disc Degeneration"[Mesh] OR "Intervertebral Disc Degeneration"[Mesh] ) | 28,265 |
| #2 | (((((((((((((((Disc Displacement, Intervertebral) OR (Intervertebral Disc Displacements)) OR (Protruded Disc)) OR (Disc, Protruded)) OR (Discs, Protruded)) OR (Protruded Discs)) OR (Protruded Disk)) OR (Intervertebral Disk Displacement)) OR (Disk Displacement, Intervertebral)) OR (Intervertebral Disk Displacements)) OR (Herniated Disks)) OR (Slipped Disk)) OR (Disk Prolapses)) OR (Prolapsed Disk)) OR (Herniated Disc)) OR (Herniated Discs) | 23,406 |
| #3 | #1 OR #2 | 30,282 |
| #4 | "manipulation, osteopathic"[MeSH Terms] OR "osteopathic manipulation"[Title/Abstract] OR "osteopathic treat*"[Title/Abstract] OR "OMT"[Title/Abstract] OR "manipulation, chiropractic"[MeSH Terms] OR "chiropractic manipulation"[Title/Abstract] OR "chiropractic adjustment"[Title/Abstract] OR "chiropractic care"[Title/Abstract] OR "manipulation, orthopedic"[MeSH Terms] OR "thrust*"[All Fields] OR "manipulation, spinal"[MeSH Terms] OR "spinal manipulat*"[All Fields] OR "spine manipulat*"[All Fields] OR "cervical manipulat*"[All Fields] OR "lumbar manipulat*"[All Fields] OR "spinal adjust*"[All Fields] OR "high-velocity low-amplitude"[All Fields] OR "high-velocity"[All Fields] OR "lowamplitude"[All Fields] OR "HLVA"[All Fields] OR "HVLA-SM"[All Fields] | 19,770 |
| #5 | (((((((Chiropractic Manipulation) OR (Spinal Adjustment, Chiropractic)) OR (Adjustment, Chiropractic Spinal)) OR (Adjustments, Chiropractic Spinal)) OR (Chiropractic Spinal Adjustment)) OR (Chiropractic Spinal Adjustments)) OR (Spinal Adjustments, Chiropractic)) OR (Chiropractic Adjustment) | 3,114 |
| #6 | #4 OR #5 | 20,860 |
| #7 | #3 AND #6 | 371 |
| #8 | (("Brain Mapping"[Mesh]) OR "Functional Neuroimaging"[Mesh]) OR "Neuroimaging"[Mesh] | 208,170 |
| #9 | ((((((Neuroimaging, Functional) OR (Functional Brain Imaging)) OR (Brain Imaging, Functional)) OR (Brain Imagings, Functional)) OR (Functional Brain Imagings)) OR (Functional Cerebral Localization)) OR (Functional Cerebral Localizations) | 219,853 |
| #10 | #8 OR #9 | 306,400 |
| #11 | ("Pain"[Mesh]) OR "Low Back Pain"[Mesh] | 491,294 |
| #12 | (((((((Back Pain, Low) OR (Back Pains, Low)) OR (Low Back Pains)) OR (Pain, Low Back)) OR (Lumbago)) OR (Recurrent Low Back Pain)) OR (Low Back Pain, Posterior Compartment)) OR (Low Back Aches) OR (Suffering, Physical) OR (Aches) | 1,153,973 |
| #13 | #11 OR #12 | 1,153,973 |
| #14 | #10 AND #13 | 11,578 |
| #15 | #7 AND #14 | 10 |

Cochrane Library Search Results Information Table

| **Number** | **Search Expression** | **Number of Results** |
| --- | --- | --- |
| #1 | MeSH descriptor: [intervertebral Disc Displacement] explode all trees | 1401 |
| #2 | (protrud* disc* OR disc* protrus* OR intervertebral disk* hemiat* OR disk* protrus* OR intervertebral disk* displacement* OR prolaps* disk* oR disc* hemiat OR intervertebral disk* protrus*):ti,ab,kw | 1336 |
| #3 | #1 OR #2 | 2029 |
| #4 | MeSH descriptor: [Musculoskeletal Manipulations] explode all trees | 4399 |
| #5 | (manipulation osteopathic OR osteopathic treat* OR OMT OR manipulation chiropractic OR chiropractic adjustment OR chiropractic care OR manipulation orthopedic OR thrust* OR manipulation spinal OR spinal manipulat* OR spine manipulat* OR cervical manipulat* OR lumbar manipulat* OR spinal adjust* OR high-velocity low-amplitude OR high-velocity OR low-amplitude OR HLVA OR HVLA-SM OR bodywork* OR rolfing OR musculoskeletal manipulaion* OR manual therap* OR reflexology OR craniosacral massage* OR manipulation therap*):ti,ab,kw | 18316 |
| #6 | #4 OR #5 | 21021 |
| #7 | #3 AND #6 | 111 |
| #8 | MeSH descriptor: [Functional Neuroimaging] explode all trees | 2722 |
| #9 | (functional brain imaging* OR functional neuroimaging*):ti,ab,kw | 9641 |
| #10 | #8 OR #9 | 10751 |
| #11 | MeSH descriptor: [Pain] explode all trees | 73563 |
| #12 | MeSH descriptor: [Low Back Pain] explode all trees | 6321 |
| #13 | (low back pain* oR lower back pain* oR lumbago OR low backache* OR (mechanical NEAR/3 low back pain) OR (postural NEAR/3 low back pain) oR recurrent low back pain):ti,ab,kw | 19230 |
| #14 | #11 OR #12 OR #13 | 84932 |
| #15 | #10 AND #14 | 355 |
| #16 | #7 AND #15 | 1 |

Embase Search Results Information Table

| **Number** | **Search Expression** | **Number of Results** |
| --- | --- | --- |
| #1 | 'lumbar disk hernia'/exp | 10872 |
| #2 | 'discal lumbosciatica' OR 'disk hernia, lumbar' OR 'displaced lumbar disc' OR 'hernia disci lumbalis' OR 'hernia, lumbar disk' OR 'herniated lumbar disc' OR 'herniated lumbar disk' OR 'herniated lumbar intervertebral disc' OR 'herniated lumbar intervertebral disk' OR 'lumbar disc displacement' OR 'lumbar disc hernia' OR 'lumbar disc herniation' OR 'lumbar disc prolapse' OR 'lumbar disc protrusion' OR 'lumbar disc rupture' OR 'lumbar disc syndrome' OR 'lumbar discal hernia' OR 'lumbar discal herniation' OR 'lumbar discal prolapse' OR 'lumbar discal protrusion' OR 'lumbar disk herniation' OR 'lumbar disk prolapse' OR 'lumbar disk protrusion' OR 'lumbar disk syndrome' OR 'lumbar intervertebral disc displacement' OR 'lumbar intervertebral disc hernia' OR 'lumbar intervertebral disc herniation' OR 'lumbar intervertebral disk hernia' OR 'lumbar intervertebral disk herniation' OR 'lumbar intervertebral prolapse' OR 'lumbar vertebral disc hernia' OR 'lumbar vertebral disc herniation' OR 'lumbar vertebral disk hernia' OR 'lumbar vertebral disk herniation' OR 'median lumbar disk prolapse' OR 'plid (prolapse)' OR 'prolapse of lumbar intervertebral disc' OR 'prolapsed lumbar intervertebral disc' OR 'prolapsed lumbar intervertebral disk' OR 'protruded lumbar intervertebral disc' OR 'protruded lumbar intervertebral disk' OR 'slipped lumbar disc' OR 'slipped lumbar disk' OR 'lumbar disk hernia' | 13868 |
| #3 | #1 OR #2 | 13868 |
| #4 | 'osteopathic manipulation'/exp OR 'osteopathic manipulation' OR 'osteopathic manipulation':ti,ab,kw OR 'osteopathic treat*':ti,ab,kw OR 'omt':ti,ab,kw OR 'chiropractic manipulation'/exp OR 'chiropractic manipulation' OR 'chiropractic manipulation':ti,ab,kw  OR 'chiropractic adjustment':ti,ab,kw OR 'chiropractic care':ti,ab,kw OR 'orthopedic manipulation'/exp OR 'orthopedic manipulation' OR 'thrust*' OR 'spine manipulation'/exp OR 'spine manipulation' OR 'spinal manipulat*' OR 'spine manipulat*' OR 'cervical manipulat*' OR 'lumbar manipulat*' OR 'spinal adjust*' OR 'high-velocity low-amplitude' OR 'high-velocity' OR 'low-amplitude' OR 'hlva' OR 'hvla-sm | 6483 |
| #5 | 'musculoskeletal manipulations' OR 'musculoskeletal manipulation' | 1068 |
| #6 | #4 OR #5 | 6729 |
| #7 | #3 AND #6 | 92 |
| #8 | 'neurologic examination'/exp | 830273 |
| #9 | 'brain diagnosis' OR 'diagnostic techniques, neurological' OR 'neuro-diagnosis' OR 'neuro-examination' OR 'neurodiagnosis' OR 'neuroexamination' OR 'neurologic diagnosis' OR 'neurological diagnosis' OR 'neurological diagnostic techniques' OR 'neurological examination' OR 'neurologic examination' | 104042 |
| #10 | #8 OR #9 | 838512 |
| #11 | 'pain'/exp | 1994501 |
| #12 | 'acute pain' OR 'deep pain' OR 'lightning pain' OR 'nocturnal pain' OR 'pain response' OR 'pain syndrome' OR 'treatment related pain' OR 'pain' | 1960567 |
| #13 | #11 OR #12 | 2463113 |
| #14 | #10 AND #13 | 88027 |
| #15 | #4 AND #14 | 6 |

Web of Science Search Results Information Table

| **Number** | **Search Expression** | **Number of Results** |
| --- | --- | --- |
| #1 | ((((((((((((TS=(lumbar disk hernia))) OR ALL=(discal lumbosciatica)) OR ALL=(disk hernia, lumbar)) OR ALL=(displaced lumbar disc)) OR ALL=(hernia disci lumbalis)) OR ALL=(hernia, lumbar disk)) OR ALL=(herniated lumbar disc)) OR ALL=(herniated lumbar disk)) OR ALL=(herniated lumbar intervertebral disc)) OR ALL=(herniated lumbar intervertebral disk)) OR ALL=(lumbar disc displacement)) OR ALL=(lumbar disc hernia) | 3751 |
| #2 | ((TS=(musculoskeletal manipulation)) OR ALL=(musculoskeletal manipulations)) OR ALL=(musculoskeletal manipulation) | 2462 |
| #3 | #1 AND #2 | 7 |
| #4 | ((((((((TS=(neurologic examination)) OR ALL=(brain diagnosis)) OR ALL=(diagnostic techniques, neurological)) OR ALL=(neuro-diagnosis)) OR ALL=(neuro-examination)) OR ALL=(neurodiagnosis)) OR ALL=(neuroexamination)) OR ALL=(neurologic diagnosis)) OR ALL=(neurological diagnosis) | 229592 |
| #5 | $(((((TS=(pain)$ OR ALL=(acute pain)) OR ALL=(deep pain)) OR ALL=(lightning pain)) OR ALL=(nocturnal pain)) OR ALL=(pain response)) OR ALL=(Low Back Pain) | 937609 |
| #6 | #4 AND #5 | 12647 |
| #7 | #3 AND #6 | 1 |

Chinese Database Literature Retrieval Information Table

| **Database** | **Search Strategy** | **Number of Search Results** |
| --- | --- | --- |
| China National Knowledge Infrastructure (CNKI) | ((TKA=（推拿+按摩+脊柱推拿+手法治疗）OR SU=（推拿+按摩+脊柱推拿+手法治疗）) AND (TKA=（腰椎间盘突出症+腰椎间盘突出+椎间盘移位+腰椎间盘退变）OR SU=（椎间盘移位+腰椎间盘突出+椎间盘移位+腰椎间盘退变）)) AND ((TKA=（疼痛+痛感+情绪+疼+痛）OR SU=（疼痛+痛感+情绪+疼+痛）) AND (TKA=（fMRI+磁共振成像+磁共振+神经影像学+脑）OR SU=（fMRI+磁共振成像+磁共振+神经影像学+脑）)) | 54 |
| Wanfang Database | （(主题:(推拿) or 题名或关键词:(手法治疗 or 脊柱推拿 or 按摩)) and （主题:(椎间盘移位) or 题名或关键词:(腰椎间盘突出 or 椎间盘移位 or 腰椎间盘退变)）） and （（主题:(疼痛) or 题名或关键词:(痛感 or 情绪 or 疼 or 痛)） and （主题:(fMRI) or 题名或关键词:(磁共振成像 or 磁共振 or 神经影像学 or 脑)）） | 66 |
| VIP Chinese Journal Service Platform (VIP) | （M=(推拿 OR 手法治疗 OR 脊柱推拿 OR 按摩) AND M=(腰椎间盘突出症 OR 椎间盘突出 OR 椎间盘移位 OR 腰椎间盘退变)） AND （M=(疼痛 OR 痛感 OR 情绪 OR 疼 OR 痛) AND M=(fMRI OR 磁共振成像 OR 磁共振 OR 神经影像学 OR 脑)） | 7 |
| Chinese Biomedical Literature Service System (SinoMed) | #1: "椎间盘移位"[不加权:扩展] #2 : "椎间盘脱出;"[常用字段:智能] OR ""[常用字段:智能] OR "突出椎间盘;"[常用字段:智能] OR ""[常用字段:智能] OR "椎间盘突出;"[常用字段:智能] OR ""[常用字段:智能] OR "椎间盘移位;"[常用字段:智能] AND ""[常用字段:智能] OR "椎间盘脱出"[常用字段:智能] #3: ("椎间盘脱出;"[常用字段:智能] OR ""[常用字段:智能] OR "突出椎间盘;"[常用字段:智能] OR ""[常用字段:智能] OR "椎间盘突出;"[常用字段:智能] OR ""[常用字段:智能] OR "椎间盘移位;"[常用字段:智能] AND ""[常用字段:智能] OR "椎间盘脱出"[常用字段:智能]) OR ("椎间盘移位"[不加权:扩展]) #4 "推拿疗法"[不加权:扩展] AND "推拿疗法"[不加权:扩展] #5 ("推拿疗法"[不加权:扩展] AND "推拿疗法"[不加权:扩展]) AND (("椎间盘脱出;"[常用字段:智能] OR ""[常用字段:智能] OR "突出椎间盘;"[常用字段:智能] OR ""[常用字段:智能] OR "椎间盘突出;"[常用字段:智能] OR ""[常用字段:智能] OR "椎间盘移位;"[常用字段:智能] AND ""[常用字段:智能] OR "椎间盘脱出"[常用字段:智能]) OR ("椎间盘移位"[不加权:扩展])) #6 ("腰痛"[不加权:扩展]) OR "疼痛"[不加权:扩展] #7 "磁共振成像"[不加权:扩展] #8 "NMR断层照相术"[常用字段:智能] OR "断层摄影术"[常用字段:智能] OR "核磁共振成像"[常用字段:智能] OR "MR断层摄影术"[常用字段:智能] OR "fMRI"[常用字段:智能] OR "MRI"[常用字段:智能] #9 ("NMR断层照相术"[常用字段:智能] OR "断层摄影术"[常用字段:智能] OR "核磁共振成像"[常用字段:智能] OR "MR断层摄影术"[常用字段:智能] OR "fMRI"[常用字段:智能] OR "MRI"[常用字段:智能]) OR ("磁共振成像"[不加权:扩展]) #10 ((("NMR断层照相术"[常用字段:智能] OR "断层摄影术"[常用字段:智能] OR "核磁共振成像"[常用字段:智能] OR "MR断层摄影术"[常用字段:智能] OR "fMRI"[常用字段:智能] OR "MRI"[常用字段:智能]) OR ("磁共振成像"[不加权:扩展])) AND (("腰痛"[不加权:扩展]) OR "疼痛"[不加权:扩展]) #11 (((("NMR断层照相术"[常用字段:智能] OR "断层摄影术"[常用字段:智能] OR "核磁共振成像"[常用字段:智能] OR "MR断层摄影术"[常用字段:智能] OR "fMRI"[常用字段:智能] OR "MRI"[常用字段:智能]) OR ("磁共振成像"[不加权:扩展])) AND (("腰痛"[不加权:扩展]) OR "疼痛"[不加权:扩展])) AND (("推拿疗法"[不加权:扩展] AND "推拿疗法"[不加权:扩展]) AND (("椎间盘脱出;"[常用字段:智能] OR ""[常用字段:智能] OR "突出椎间盘;"[常用字段:智能] OR ""[常用字段:智能] OR "椎间盘突出;"[常用字段:智能] OR ""[常用字段:智能] OR "椎间盘移位;"[常用字段:智能] AND ""[常用字段:智能] OR "椎间盘脱出"[常用字段:智能]) OR ("椎间盘移位"[不加权:扩展]))) | #1: 71000 #2: 92356 #3: 92356 #4: 60595 #5: 3148 #6: 575037 #7: 704026 #8: 957304 #9: 957304 #10: 19804 #11: 24 |
